# Prevalence of depressive symptoms among Chinese university students amid the COVID-19 pandemic: a systematic review and meta-analysis

**DOI:** 10.1017/S2045796021000202

**Published:** 2021-03-26

**Authors:** Wei Luo, Bao-Liang Zhong, Helen Fung-Kum Chiu

**Affiliations:** 1 Research Center for Psychological and Health Sciences, China University of Geosciences, Wuhan, China; 2 Affiliated Wuhan Mental Health Center, Tongji Medical College of Huazhong University of Science & Technology, Wuhan, China; 3 Department of Psychiatry, The Chinese University of Hong Kong, Hong Kong SAR, China

**Keywords:** COVID-19, depressive symptoms, meta-analysis, prevalence, systematic review, university students

## Abstract

**Aims:**

Chinese university students are at high risk for depressive symptoms and the ongoing coronavirus disease 2019 (COVID-19) pandemic may have exacerbated the mental health of university students. However, existing studies on depressive symptoms in Chinese university students during the COVID-19 pandemic reported a wide range of prevalence estimates, making mental health planning for this population difficult. The objective of this study was to conduct a systematic review and meta-analysis of surveys that assessed the prevalence of depressive symptoms in Chinese university students amid the COVID-19 pandemic.

**Methods:**

Major Chinese (CNKI, Wanfang, VIP) and English (PubMed, Embase, PsycInfo) databases and preprint platforms were searched to identify cross-sectional studies containing data on the prevalence of depressive symptoms in Chinese university students during the pandemic. Two authors independently retrieved the literature, evaluated the eligibility of potential studies, assessed the risk of bias (RoB) of included studies, and extracted data. RoB was assessed with the Joanna Briggs Institute Critical Appraisal Checklist for Studies Reporting Prevalence Data.

**Results:**

In total, 1177 records were retrieved, and 84 studies involving 1 292 811 Chinese university students during the pandemic were included. None of the included studies were rated as completely low RoB. Statistically significant heterogeneity in the prevalence estimates of included studies was detected (*I*^2^ = 99.9%, *p* < 0.001). The pooled prevalence of depressive symptoms was 26.0% (95%CI: 23.3–28.9%), which was significantly higher in female than in male students (30.8% *v*. 28.6%, *p* < 0.001), in postgraduates than in undergraduates (29.3% *v*. 22.9%, *p* < 0.001), in students living inside than in those living outside the COVID-19 epicentre (27.5% *v*. 22.3%, *P* < 0.001), in students from universities at the epicentre than in those from universities outside the epicentre (26.2% *v*. 23.1%, *p* < 0.001), in students who had close contact with COVID-19 than in those who did not (46.0% *v*. 25.0%, *p* < 0.001), and in students who had acquaintances or relatives infected with COVID-19 (39.7% *v*. 24.0%, *p* < 0.001) than in those who did not. Five sources of heterogeneity were identified from the subgroup analysis: survey period, % of males among the survey sample, scale of depressive symptoms, cutoff score of the scale and level of RoB.

**Conclusions:**

Over one-fourth of Chinese university students experienced depressive symptoms during the COVID-19 pandemic. Mental health services for this population should include periodic evaluation of depressive symptoms, expanded social support and psychiatric assessment and treatment when necessary. It is also necessary to design depression prevention programmes that target higher-risk cohorts of university students.

## Introduction

Studying in university is an important life stage during which a person moves from family dependence to independence and socialisation. The transition is challenging because of the high level of academic and employment stress and the prevalent interpersonal, romantic and emotional problems in this particular stage for university students (Zhao *et al*., 2015; Liu *et al*., 2017; Zhang *et al*., 2020*a*). However, due to China's strict examination-oriented education system, many university students have little training in interpersonal communication, problem solving and teamwork skills before entering university. Therefore, this population has difficulties in adapting to the university environment and is more likely to feel unconfident and confused about the future (Kirkpatrick and Zang, 2011; Hu, 2018). Moreover, university students in China have a high likelihood of experiencing parent−adolescent conflict owing to the popular authoritarian parenting style in the context of Chinese culture, which is characterised by high control and high warmth (Marmorstein and Iacono, 2004; Diao, 2007; Ren and Edwards, 2015). As a result, Chinese university students are at high risk for common mental health problems; for example, empirical evidence from a systematic review of 39 studies has shown that as high as 23.8% of Chinese university students suffer from depressive symptoms (Lei *et al*., 2016).

The ongoing coronavirus disease 2019 (COVID-19) pandemic has caused a global mental health crisis. Lessons learned from the 2003 severe acute respiratory syndrome (SARS) epidemic in China suggest that depressive symptoms are one of the most common mental health problems among university students; for example, during the SARS epidemic, 25.4–29.6% of the Chinese university students had depressive symptoms (Dang *et al*., 2004; Liu *et al*., 2004). In China, the pandemic has changed many aspects of university students’ daily lives. Despite an increase in time spent with parents, home-isolated students have an increased chance of conflicting with parents (Luo, 2020). To prevent the spread of the epidemic, students are not allowed to return to campus to resume their studies, potentially delaying their graduation dates. Furthermore, because of social distancing and stay-at-home requirements, social and peer interactions are reduced, likely resulting in an increased level of social disconnectedness and a decreased level of peer support. Because parent−adolescent conflict, social disconnectedness and a lack of peer support have been associated with depressive symptoms in adolescents (Vaughan *et al*., 2010; Elmer and Stadtfeld, 2020; Rognli *et al*., 2020), the emotional health of Chinese university students may have been exacerbated by the COVID-19 pandemic.

Mental health services and crisis psychological intervention have been an essential part of the battle against the COVID-19 pandemic (Li *et al*., 2020*a*). To facilitate the development of population-specific intervention programmes, it is necessary to understand the epidemiology of depressive symptoms in university students in China amid the COVID-19 pandemic. However, available studies on depressive symptoms among Chinese university students have varied widely in terms of sampling methods, sample sizes and assessments of depressive symptoms, and most importantly, there have been considerable variations in the reported prevalence of depressive symptoms (1.8–79.3%) (Liang *et al*., 2020*a*; Ren *et al*., 2020*b*), making mental health policy-making and planning difficult. To help clarify this issue, we performed a systematic review and meta-analysis on the prevalence of depressive symptoms among Chinese university students during the COVID-19 pandemic.

## Methods

This systematic review and meta-analysis was reported in accordance with the Preferred Reporting Items for Systematic Reviews and Meta-Analyses (PRISMA) guidelines, and the protocol was registered in the International Prospective Register of Systematic Reviews (PROSPERO) with the registration number CRD 42020206666.

### Inclusion and exclusion criteria

The inclusion criteria for eligible studies were (a) cross-sectional surveys or baseline surveys of cohort studies with meta-analysable data (i.e. reporting the prevalence of depressive symptoms); (b) study subjects were Chinese university students, including overseas students and postgraduates; (c) the presence of depressive symptoms was assessed with standardised instruments and (d) the study was conducted during the COVID-19 pandemic (since 1 January 2020). We excluded studies with mixed samples that did not present results separately for university students and studies that assessed depressive symptoms with unstandardised instruments (i.e. a simple self-designed question or a self-designed scale without convincing evidence of reliability and validity).

### Literature search

We searched potential studies published between 1 January 2020 and 10 February 2021 in both Chinese and English bibliographic databases: China National Knowledge Infrastructure, Wanfang data, VIP Information, PubMed, Embase and PsycInfo. Key terms used were: (adolescen* OR teenager* OR youth* OR student* OR young adult* OR undergraduate* OR universit* OR college*), (coronavirus disease 2019 or severe acute respiratory syndrome coronavirus 2 or COVID-19 or COVID) and (depress*). To avoid missing relevant studies, reference lists of the retrieved reviews and included studies were also hand-searched. Preprint servers were also searched to retrieve grey literature: medRxiv, bioRxiv, PsyArXiv, ChinaXiv and Research Square. The literature search was ended on 12 February 2021. Detailed search strategies are provided in online Supplementary Table 1.

### Data extraction

By using a predesigned electronic form, the following variables were extracted from included studies: first author, study site, study period, characteristics of the study sample, sampling method, sample size, survey method, assessment of depressive symptoms and rates of depressive symptoms. According to the State Council Information Office of the People's Republic of China (The State Council Information Office of the People's Republic of China, 2020), the study period in China was roughly classified as early stage of the COVID-19 outbreak (20 January–20 February 2020), late stage of the COVID-19 outbreak (21 February–28 April 2020) and post-COVID-19 outbreak (since 29 April 2020).

### RoB assessment of included studies

We used the Joanna Briggs Institute (JBI) Critical Appraisal Checklist for Studies Reporting Prevalence Data (abbreviated as ‘JBI checklist’ hereafter) to assess the RoB of included studies (Munn *et al*., 2014). This checklist evaluates the RoB in terms of nine methodological domains: sample frame, sampling, sample size, description of subjects and setting, sample coverage of the data analysis, validity of the method for assessing the outcome, standardisation and reliability of the method for assessing outcome, statistical analysis and response rate. Two example items of the JBI checklist used in the current study were ‘Was the sample size adequate?’ and ‘Were valid methods used for assessing depressive symptoms?’. Each item has four choices: yes, no, unclear or not applicable. One point is assigned to a ‘yes’ response, and the RoB score is the sum of the nine items, ranging from zero to nine, with a higher score indicating a lower RoB. In this study, the level of RoB of included studies was operationally categorised into low (RoB score of ‘7–9’), moderate (RoB score of ‘4–6’) and high (RoB score of ‘0–3’). A RoB score of nine represents ‘completely low RoB’.

Literature search, study inclusion, data extraction and RoB assessment were independently performed by the first and second authors of this study. They discussed their differences to arrive at a consensus when disagreement occurred in an assessment.

### Statistical analysis

We used meta-analysis to generate pooled estimates and their 95% confidence intervals (95%CIs) for the prevalence of depressive symptoms in the whole sample and in various cohorts of the sample. Forest plots were adopted to display the prevalence rates and pooled estimates. We used the *I*^2^ test to evaluate heterogeneity between studies. When there was little evidence of heterogeneity (i.e. *I*^2^ ⩽ 50%, heterogeneity *P* ⩾ 0.10), a fixed-effect model was used to generate the pooled estimates; otherwise, the random-effect model was used. The pooled rates of various cohorts were compared by using the *Z* test. We used subgroup analysis to explore the source of heterogeneity in the prevalence estimate of depressive symptoms. The *Q*-value test was used to test the significance of differences in prevalence rates between subgroups. Publication bias was assessed with funnel plots and Begg's test, since Begg's test is fairly powerful for large meta-analyses that include 75 or more original studies (Begg and Mazumdar, 1994). Before pooled analysis, prevalence proportions were transformed by using the Freeman−Tukey variant of the arcsine square root, Arcsine, untransformed, Log or Logit, as appropriate (Barendregt *et al*., 2013). All analyses were conducted using R (version 4.0.2). A two-sided *P* < 0.05 was considered statistically significant.

## Results

### Characteristics of included studies

**Fig. 1.**
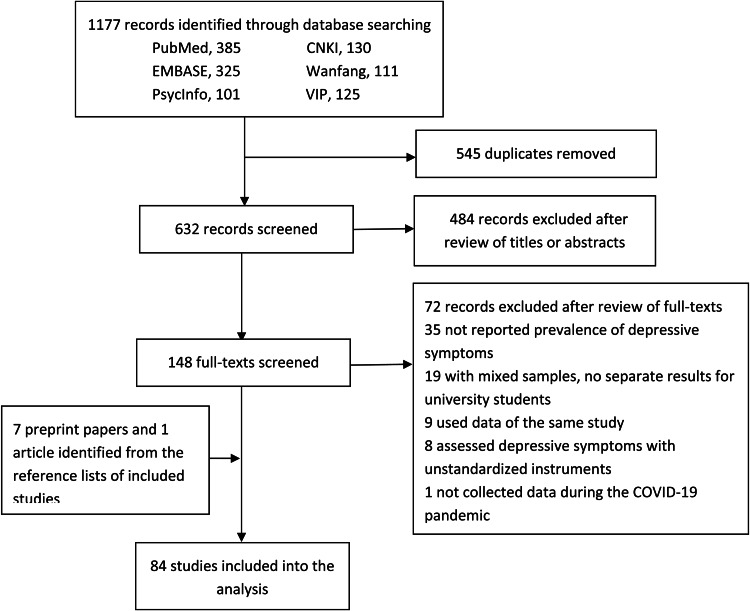
Flowchart of study inclusion.

The process of study inclusion is shown in Fig. 1. Finally, this meta-analysis included 84 studies with a total of 1 292 811 Chinese university students (Cao, 2020; Chang *et al*., 2020; Chen *et al*., 2020*a*, 2020*b*, 2020*c*, 2020*d*; Chi *et al*., 2020; Cong *et al*., 2020; Deng *et al*., 2020; Dong, 2020; Dong *et al*., 2020; Feng, 2020; Feng *et al*., 2020; Han *et al*., 2020; Ji *et al*., 2020; Jiang *et al*., 2020; Lei *et al*., 2020; Li and He, 2020; Li *et al*., 2020*b*; Lian *et al*., 2020; Liang *et al*., 2020*a*, 2020*b*; Lin and Xu, 2020; Lin *et al*., 2020*a*, 2020*b*; Liu, 2020*a*, 2020*b*; Liu *et al*., 2020*a*, 2020*b*, 2020*c*; Ma *et al*., 2020*a*, 2020*b*; Mao *et al*., 2020; Qian, 2020; Ren *et al*., 2020*a*, 2020*b*; 2020*c*; Si *et al*., 2020; Sun *et al*., 2020, 2021; Tang *et al*., 2020; Wan and Shao, 2020; Wang and He, 2020; Wang and Li, 2020; Wang *et al*., 2020*b*; 2020*c*; 2020*d*; 2020*e*; 2020*f*; 2021; Wei, 2020; Wu *et al*., 2020, 2021; Xiang *et al*., 2020; Xiao *et al*., 2020*a*, 2020*b*; Xie *et al*., 2020; Xin *et al*., 2020; Xing *et al*., 2020; Xiong *et al*., 2020; Xu and Li, 2020; Yan *et al*., 2020; Yang *et al*., 2020*b*; Yao *et al*., 2020; Yi *et al*., 2020*a*, 2020*b*; Yu *et al*., 2020, 2021; Zhan *et al*., 2020; Zhang *et al*., 2020*b*, 2020*c*, 2020*d*, 2020*e*, 2020*f*, 2020*g*; 2020*h*; Zhao and Hu, 2020; Zhao *et al*., 2020*a*, 2020*b*, 2020*c*; Zhou *et al*., 2020; Chen and Zhu, 2021; Ni *et al*., 2021; Pan *et al*., 2021). Among the 84 studies, seven were preprint articles (Cong *et al*., 2020; Liu *et al*., 2020*c*; Si *et al*., 2020; Xiong *et al*., 2020; Zhang *et al*., 2020*h*; Zhao *et al*., 2020*b*; Zhou *et al*., 2020), eight had samples recruited from universities at China's COVID-19 epicentre (Hubei or Wuhan) (Deng *et al*., 2020; Liu *et al*., 2020*a*; Wang *et al*., 2020*d*, 2020*e*; Xiao *et al*., 2020*b*, 2020*a*; Xu and Li, 2020; Wu *et al*., 2021) and two recruited samples of overseas Chinese students (Cong *et al*., 2020; Zhao *et al*., 2020*b*). A total of 23 studies adopted probability sampling to recruit subjects, while the remaining studies adopted convenience sampling. The sample sizes of included studies ranged between 84 and 746 217, with a median of 973. A vast majority of the studies collected data via online self-administered questionnaires, while seven collected data via paper−pencil self-administered questionnaires (Chen *et al*., 2020*a*, 2020*c*, 2020*d*; Dong *et al*., 2020; Liu, 2020*b*; Liu *et al*., 2020*b*; Wu *et al*., 2020). Among the included studies, the Nine-item Patient Health Questionnaire (PHQ-9) was the most common instrument to assess the presence of depressive symptoms (*n* = 37), followed by Zung's Self-rating Depression Scale (SDS) (*n* = 22), the depression subscale of the Symptom Checklist-90-Revised (SCL-90-R) (*n* = 8), the depression subscale of the Depression, Anxiety and Stress Scale – 21 Items (DASS-21) (*n* = 7) and the Center for Epidemiologic Studies – Depression Scale (CES-D) (*n* = 7). The average and median reported prevalence rates of depressive symptoms were 27.3% and 25.8%, respectively. Other detailed characteristics of the included studies are shown in Table 1.

**Table 1.** Characteristics of included studies

Study	Subjects and setting	Dates of the survey	Sampling method	Sample size	Male students, *n* (%)	Age (years)	Survey method	Assessment of depressive symptoms	Depressed students, *n* (%)
Cao (2020)	Undergraduates of a junior college in Xi'an, China	NR	Convenience sampling	2733	1684 (61.6)	Range: 16–24	Online self-administered questionnaire	PHQ-9 ⩾ 5	575 (21.0)
Chang *et al*. (2020)	University students in Guangdong, China	31 January–3 February 2020	Convenience sampling	3881	1434 (36.9)	Mean: 20.0	Online self-administered questionnaire	PHQ-9 ⩾ 5	821 (21.2)
Chen *et al*. (2020*a*)	Medical postgraduates of a general hospital in Hangzhou, China	February 2020	Convenience sampling	795	343 (43.1)	Mean: 26.6	Paper−pencil self-administered questionnaire	PHQ-9 ⩾ 5	172 (21.6)
Chen *et al*. (2020*b*)	Undergraduates of 85 universities in Guangdong, China	13–22 February 2020	Convenience sampling	323 489	130 516 (40.3)	⩽18: 29 510 (9.1) 19–20: 167 932 (51.9) 21–22: 104 800 (32.4) 23–24: 19 710 (6.1) ⩾25: 1537 (0.5)	Online self-administered questionnaire	PHQ-9 ⩾ 10	24 909 (7.7)
Chen *et al*. (2020*c*)	Medical postgraduates of a general hospital in Hangzhou, China	3–16 February 2020	Cluster sampling	286	NR	NR	Paper−pencil self-administered questionnaire	PHQ-9 ⩾ 5	54 (18.9)
Chen *et al*. (2020*d*)	Undergraduates and postgraduates in Beijing, China	‘post-epidemic of CPVID-19’	Stratified random sampling	697	183 (26.3)	Mean: 24.3	Paper−pencil self-administered questionnaire	Depression subscale of SCL-90-R > 2	61 (8.8)
Chi *et al*. (2020)	University students in China	12–17 February 2020	Convenience sampling	2038	755 (37.0)	Mean: 20.6	Online self-administered questionnaire	PHQ-9 ⩾ 10	475 (23.3)
Cong *et al*. (2020)	Oversea Chinese undergraduates and postgraduates	May 18–21, 2020	Convenience sampling	252	102 (40.5)	<18: 2 (0.8) 18–25: 160 (63.5) 26–30: 70 (27.8) >30: 20 (7.9)	Online self-administered questionnaire	PHQ-9 ⩾ 5	152 (60.3)
Deng *et al*. (2020)	Undergraduates in China	8–11 May 2020	Convenience sampling	1607	1041 (64.8)	<18: 20(1.2) 18–22: 1573 (97.9) >22: 14 (0.9)	Online self-administered questionnaire	Depression subscale of DASS21 ⩾ 10	56 (3.5)
Dong *et al*. (2020)	Medical postgraduates of a general hospital in China	20 January–20 February 2020	Convenience sampling	162	52 (32.1)	Mean: 26.4	Paper−pencil self-administered questionnaire	SDS ⩾ 53	63 (38.9)
Dong (2020)	Undergraduates of a university in Linfen, China	NR	Cluster sampling	4085	923 (22.6)	Mean: 18.9	Online self-administered questionnaire	Depression subscale of SCL-90-R > 2	554 (13.6)
Feng *et al*. (2020)	Students of a university in Beijing, China	8–28 February 2020	Simple cluster sampling	1346	364 (27.0)	Mean: 19.8	Online self-administered questionnaire	PHQ-9 ⩾ 5	429 (31.9)
Feng (2020)	Undergraduates of a junior college in Qingyuan, China	18–22 February 2020	Random sampling	7157	2158 (30.2)	Median: 20.1	Online self-administered questionnaire	PHQ-9 ⩾ 5	1956 (27.3)
Han *et al*. (2020)	Undergraduates and postgraduates in China	22–24 February 2020	Convenience sampling	405	134 (33.1)	NR	Online self-administered questionnaire	Depression subscale of DASS21 ⩾ 10	178 (44.0)
Ji *et al*. (2020)	Nursing undergraduates of seven universities in Sichuan, China	14–19 February 2020	Cluster sampling	1013	139 (13.7)	Mean: 20.0	Online self-administered questionnaire	SDS ⩾50	247 (24.4)
Jiang *et al*. (2020)	Medical undergraduates of a university in China	27–29 February 2020	Cluster sampling	399	162 (40.6)	NR	Online self-administered questionnaire	PHQ-9 ⩾ 5	104 (26.1)
Lei *et al*. (2020)	Medical undergraduates and postgraduates of a university in Tangshan, China	NR	Convenience sampling	231	109 (47.2)	NR	Online self-administered questionnaire	SDS ⩾ 53	143 (61.9)
Li and He (2020)	Students of a junior college in Jinhua, China	30 January–15 February 2020	Convenience sampling	1144	597 (52.2)	NR	Online self-administered questionnaire	PHQ-9 ⩾ 5	240 (21.0)
Li *et al*. (2020*b*)	Undergraduates of a university in Chengdu, China	February–March 2020	Cluster sampling	7747	3947 (50.9)	Mean: 20.7	Online self-administered questionnaire	PHQ-9 ⩾ 5	1278 (16.5)
Lian *et al*. (2020)	Undergraduates and postgraduates of a university in Changsha, China	NR	Random sampling	1437	789 (54.9)	NR	Online self-administered questionnaire	Depression subscale of SCL-90-R ⩾ 2	177 (12.3)
Liang *et al*. (2020*a*)	Nursing junior college students and nursing undergraduates of three universities in Hebei, China	February 2020	Convenience sampling	852	80 (9.4)	NR	Online self-administered questionnaire	Depression subscale of SCL-90-R ⩾ 2	15 (1.8)
Liang *et al*. (2020*b*)	Medical postgraduates of a general hospital in Hangzhou, China	NR	Convenience sampling	793	373 (47.0)	NR	Online self-administered questionnaire	Depression subscale of SCL-90-R ⩾ 2	40 (5.0)
Lin *et al*. (2020*a*)	Undergraduates and postgraduates in China	10–16 March 2020	Convenience sampling	625	220 (35.2)	Mean: 20.2	Online self-administered questionnaire	CES-D ⩾ 16	217 (34.7)
Lin *et al*. (2020*b*)	Students of a medical university in Fuzhou, China	15–20 April 2020	Random sampling	320	149 (46.6)	NR	Online self-administered questionnaire	PHQ-9 > 5	183 (57.2)
Lin and Xu (2020)	Undergraduates of universities in Fuzhou, China	26–30 March 2020	Convenience sampling	1297	565 (43.6)	NR	Online self-administered questionnaire	PHQ-9 ⩾ 5	320 (24.7)
Liu *et al*. (2020*a*)	Undergraduates and postgraduates of a medical university in Wuhan, China	23 February—2 April 2020	Convenience sampling	217	90 (41.5)	Mean: 21.7	Online self-administered questionnaire	PHQ-9 ⩾ 5	77 (35.5)
Liu *et al*. (2020*b*)	Undergraduates of a medical university in Beijing, China	NR	Convenience sampling	611	198 (32.4)	Range: 17–23	Paper−pencil self-administered questionnaire	SDS ⩾ 53	101 (16.5)
Liu *et al*. (2020*c*)	Junior college students, undergraduates and postgraduates in China	1–5 February 2020	Convenience sampling	509	176 (34.6)	Mean: 21.3	Online self-administered questionnaire	SDS ⩾ 50	70 (13.8)
Liu (2020*a*)	Undergraduates of a university in Taiyuan, China	20–22 February 2020	Convenience sampling	191	NR	NR	Online self-administered questionnaire	Depression subscale of SCL-90-R > 2	14 (7.3)
Liu (2020*b*)	Junior college students and undergraduates in Hangzhou, China	NR	Convenience sampling	90	1 (1.1)	Range: 20–23	Paper−pencil self-administered questionnaire	SDS ⩾ 53	29 (32.2)
Ma *et al*. (2020*a*)	Undergraduates of a university in Taiyuan, China	10–15 February 2020	Random cluster sampling	516	271 (52.5)	Mean: 20.8	Online self-administered questionnaire	Depression subscale of SCL-90-R ⩾ 2	138 (26.7)
Ma *et al*. (2020*b*)	Undergraduates and postgraduates of 108 universities in Guangdong and Jiangxi, China	3–10 February 2020	Convenience sampling	746 217	331 613 (44.4)	<18: 27 640 (3.7) 18–19: 252 616 (33.9) 20–21: 327 639 (43.9) 22–23: 120 142 (16.1) 24–25: 14 925 (2.0) ⩾26: 3255 (0.4)	Online self-administered questionnaire	PHQ-9 ⩾ 7	157 452 (21.1)
Mao *et al*. (2020)	Medical postgraduates of a general hospital in Harbin, China	31 March–10 May 2020	Convenience sampling	240	124 (51.7)	Mean: 24.3	Online self-administered questionnaire	SDS ⩾ 50	93 (38.8)
Qian (2020)	Students of a medical university in Fuzhou, China	10 March 2020	Convenience sampling	535	140 (26.2)	Median: 21.0	Online self-administered questionnaire	SDS ⩾ 50	137 (25.6)
Ren *et al*. (2020*a*)	Undergraduates of a university in Zhongshan, China	NR	Convenience sampling	244	85 (34.8)	NR	Online self-administered questionnaire	Depression subscale of DASS21 ⩾ 10	78 (32.0)
Ren *et al*. (2020*b*)	Students of two universities in Inner Mongolia, China	21–28 February 2020	Convenience sampling	4560	1227 (26.9)	Mean: 21.1	Online self-administered questionnaire	SDS ⩾ 53	3614 (79.3)
Ren *et al*. (2020*c*)	Nursing junior college students and nursing postgraduates of a general hospital in Shandong, China	5–12 March 2020	Convenience sampling	294	64 (21.8)	Mean: 21.6	Online self-administered questionnaire	SDS ⩾ 50	78 (26.5)
Si *et al*. (2020)	Undergraduates and postgraduates in seven provinces in China	23 February–5 March 2020	Convenience sampling	3606	1014 (28.1)	⩽20: 1467 (40.7) 21–22: 1152 (32.0) ⩾23: 987 (27.4)	Online self-administered questionnaire	Depression subscale of DASS21 ⩾ 10	566 (15.7)
Sun *et al*. (2020)	Undergraduates in a university in Hong Kong, China	6 June 6–14 July 2020	Convenience sampling	255	33 (12.9)	Mean: 21.0	Online self-administered questionnaire	CES-D-10 ⩾ 10	145 (56.9)
Tang *et al*. (2020)	Undergraduates of six universities in Chengdu and Chongqing, China	20–27 February 2020	Convenience sampling	2485	960 (38.6)	Mean: 19.8	Online self-administered questionnaire	PHQ-9 ⩾ 10	223 (9.0)
Wan and Shao (2020)	Students of three junior colleges in Heilongjiang, China	NR	Convenience sampling	2358	1183 (50.2)	NR	Online self-administered questionnaire	PHQ-9 ⩾ 5	464 (19.7)
Wang and He (2020)	University students in Sichuan, Guizhou and Chongqing, China	Late February to middle March, 2020	Convenience sampling	1775	NR (<50)	NR	Online self-administered questionnaire	‘One SD above the mean’ on depression subscale of PQEEPH	308 (17.4)
Wang and Li (2020)	Junior college students, undergraduates and postgraduates in Sichuan, Yunnan and Chongqing, China	February 2020	Convenience sampling	3178	878 (27.6)	NR	Online self-administered questionnaire	SDS ⩾ 50	888 (27.9)
Wang *et al*. (2020*b*)	Students of a medical university in Xi'an, China	13–16 February 2020	Stratified equal proportion sampling	430	139 (32.3)	Range: 18–25	Online self-administered questionnaire	SDS ⩾53	39 (9.1)
Wang *et al*. (2020*c*)	Postgraduates in China	24 February—7 March 2020	Convenience sampling	109	38 (34.9)	20–25: 69.9 26–30: 31.5 31–40: 1.5	Online self-administered questionnaire	SDS ⩾ 53	22 (20.2)
Wang *et al*. (2020*d*)	Students of a university in Hubei, China	9–14 March 2020	Convenience sampling	2168	952 (43.9)	Mean: 20.8	Online self-administered questionnaire	CES-D ⩾ 16	752 (34.7)
Wang *et al*. (2020*e*)	Junior college students, undergraduates and postgraduates of universities in Wuhan, China	28 May–3 June 2020	Convenience sampling	3179	942 (29.6)	NR	Online self-administered questionnaire	PHQ-9 ⩾ 5	1123 (35.3)
Wang *et al*. (2020*f*)	Undergraduates and postgraduates of four universities in Guangzhou, China	31 January–5 February 2020	Cluster sampling	44 447	20 271 (45.6)	Mean: 21.0	Online self-administered questionnaire	CES-D ⩾ 28	5404 (12.2)
Wei (2020)	Students of a junior college in Guangzhou, China	13–18 February 2020	Convenience sampling	6289	NR	NR	Online self-administered questionnaire	PHQ-9 ⩾ 5	1310 (20.8)
Wu *et al*. (2020)	Undergraduates of a university in Shanghai, China	March 2020	Random sampling	807	413 (51.2)	NR	Paper−pencil self-administered questionnaire	Depression subscale of SCL-90-R ⩾ 2	216 (26.8)
Xiang *et al*. (2020)	Undergraduates and postgraduates in China	25 February–25 March 2020	Convenience sampling	1396	881 (63.1)	Mean: 20.7	Online self-administered questionnaire	SDS > 50	583 (41.8)
Xiao *et al*. (2020*a*)	Undergraduates and postgraduates of two medical universities in Beijing and Wuhan, China	4–12 February 2020	Cluster sampling	933 (Beijing: 558; Wuhan: 375)	279 (29.9)	17–24: 755 (80.9) >25: 178 (19.1)	Online self-administered questionnaire	PHQ-9 ⩾ 5	236 (25.3) Beijing: 131 (23.5) Wuhan: 105 (28.0)
Xiao *et al*. (2020*b*)	Undergraduates of two universities in Wuhan, China	5–9 February 2020	Stratified cluster sampling	3966	1591 (40.1)	NR	Online self-administered questionnaire	PHQ-9 ⩾ 5	1075 (27.1)
Xie *et al*. (2020)	Undergraduates of provinces other than Hubei in China	4–7 February 2020	Convenience sampling	2705	608 (22.5)	NR	Online self-administered questionnaire	PHQ-9 ⩾ 5	493 (18.2)
Xin *et al*. (2020)	Undergraduates and postgraduates in China	1–10 February 2020	Stratified cluster sampling	24 378	7865 (32.3)	Mean: 19.9	Online self-administered questionnaire	PHQ-9 ⩾ 10	3619 (14.8)
Xing *et al*. (2020)	Medical undergraduates of two universities in Hangzhou, China	5–7 February 2020	Convenience sampling	595	174 (29.2)	NR	Online self-administered questionnaire	PHQ-9 ⩾ 5	114 (19.2)
Xiong *et al*. (2020)	Undergraduates and postgraduates of a university in Guangzhou, China	20 February–20 March 2020	Convenience sampling	563	172 (30.6)	Mean: 21.5	Online self-administered questionnaire	Depression subscale of DASS21 ⩾ 14	69 (12.3)
Xu and Li (2020)	Undergraduates of a university in Hubei, China	18–31 May 2020	Cluster sampling	6891	2113 (30.7)	NR	Online self-administered questionnaire	SDS ⩾ 53	1874 (27.2)
Yan *et al*. (2020)	Medical undergraduates in Putian, China	23 January–23 February 2020	Cluster sampling	634	89 (14.0)	Mean: 19.3	Online self-administered questionnaire	Depression subscale of HADS > 7	146 (23.0)
Yang *et al*. (2020*b*)	Undergraduates and postgraduates of Universities in Shaanxi, China	7–9 February 2020	Convenience sampling	1667	803 (48.2)	Mean: 20.6	Online self-administered questionnaire	‘One SD above the mean’ on depression subscale of PQEEPH	257 (15.4)
Yao *et al*. (2020)	Students of a military university in China	27–28 February 2020	Convenience sampling	84	52 (61.9)	Mean: 19.9	Online self-administered questionnaire	PHQ-9 ⩾ 5	21 (25.0)
Yi *et al*. (2020*a*)	Undergraduates of a university in Zhanjiang, China	2–8 March 2020	Cluster sampling	393	121 (30.8)	Mean: 21.7	Online self-administered questionnaire	SDS ⩾ 53	104 (26.5)
Yi *et al*. (2020*b*)	Undergraduates of a medical college in Xinxiang, China	22–24 February 2020	Convenience sampling	1234	462 (37.4)	NR	Online self-administered questionnaire	PHQ-9 ⩾ 5	276 (22.4)
Yu *et al*. (2020)	Undergraduates in Guangdong, China	NR	Convenience sampling	427	98 (23.0)	NR	Online self-administered questionnaire	SDS ⩾ 53	129 (30.2)
Zhan *et al*. (2020)	Junior college students, undergraduates and postgraduates of four medical universities in Hunan and Fujian, China	17–19 March 2020	Convenience sampling	266	76 (28.6)	<18: 16 (6.0) 18–25: 188 (70.7) 26–35:58 (21.8) >35: 4 (1.5)	Online self-administered questionnaire	Depression subscale of DASS21 ⩾ 10	54 (20.3)
Zhang *et al*. (2020*b*)	Undergraduates of four universities in Guangdong, China	31 January–4 February 2020	Convenience sampling	312	67 (21.5)	Mean: 19.6	Online self-administered questionnaire	PHQ-9 ⩾ 5	92 (29.5)
Zhang *et al*. (2020*c*)	University students in China	4–7 February 2020	Convenience sampling	7833	2081 (26.6)	Mean: 19.8	Online self-administered questionnaire	PHQ-9 ⩾ 5	3053 (39.0)
Yang *et al*. (2020*a*), Zhang *et al*. (2020*d*)	Medical undergraduates in China	11–19 February 2020	Convenience sampling	6226	2484 (39.9)	Range: 18–27	Online self-administered questionnaire	PHQ-9 ⩾ 5	2206 (35.4)
Zhang *et al*. (2020*e*)	Medical undergraduates of a university in Chenzhou, China	27–29 February 2020	Cluster sampling	932	505 (54.2)	NR	Online self-administered questionnaire	PHQ-9 ⩾ 5	270 (29.0)
Zhang *et al*. (2020*f*)	Medical students of two universities in Inner Mongolia, China	February 2020	Random sampling	1486	453 (30.5)	Mean: 21.7	Online self-administered questionnaire	PHQ-9 ⩾ 5	528 (35.5)
Zhang *et al*. (2020*g*)	Undergraduates and postgraduates in China	February–April, 2020	Convenience sampling	1409	733 (52.0)	NR	Online self-administered questionnaire	SDS ⩾ 53	160 (11.4)
Zhang *et al*. (2020*h*)	Students of 57 universities in China	21–24 February 2020	Convenience sampling	2270	877 (38.6)	⩽19: 660 (29.1) 20–23: 1458 (64.2) ⩾24: 152 (6.7)	Online self-administered questionnaire	SDS > 53	237 (10.4)
Zhao *et al*. (2020*a*)	Undergraduates and postgraduates in China	23 March–20 April 2020	Convenience sampling	281	83 (29.5)	Mean: 23.6	Online self-administered questionnaire	PHQ-9 ⩾ 5	170 (60.5)
Zhao *et al*. (2020*b*)	Chinese undergraduates and postgraduates in South Korea	23 March—8 April 2020	Convenience sampling	171	57 (33.3)	Mean: 24.1	Online self-administered questionnaire	PHQ-9 ⩾ 10	49 (28.7)
Zhao *et al*. (2020*c*)	University students in China	NR	Convenience sampling	364	NR	NR	Online self-administered questionnaire	Depression subscale of DASS21 ⩾ 10	118 (32.4)
Zhao and Hu (2020)	Undergraduates of a medical college in Ganzhou, China	March–April 2020	Convenience sampling	456	240 (52.6)	Mean: 22.1	Online self-administered questionnaire	SDS ⩾ 53	50 (11.0)
Zhou *et al*. (2020)	Undergraduates and postgraduates in China	1–15 March 2020	Convenience sampling	11 133	4195 (37.7)	Median: 21.0	Online self-administered questionnaire	PHQ-9 ⩾ 5	4119 (37.0)
Chen and Zhu (2021)	Undergraduates and postgraduates of a university in Shanghai, China	12–15 March 2020	Convenience sampling	3353	1651 (49.2)	Mean: 21.8	Online self-administered questionnaire	CES-D-11 ⩾ 10	1693 (50.5)
Ni *et al*. (2021)	Medical postgraduates of a general hospital in Nanjing, China	4 March 2020	Convenience sampling	157	NR	NR	Online self-administered questionnaire	SDS ⩾ 53	76 (48.4)
Pan *et al*. (2021)	Undergraduates and postgraduates in China	4–9 March 2020	Convenience sampling	3975	1611 (40.5)	Range: 16–30	Online self-administered questionnaire	CES-D ⩾ 16	1568 (39.4)
Sun *et al*. (2021)	Undergraduates and postgraduates in China	20 March−April 2020	Convenience sampling	1912	578 (30.2)	Mean: 20.3	Online self-administered questionnaire	PHQ-9 ⩾ 5	890 (46.5)
Wang *et al*. (2021)	Junior college students, undergraduates and postgraduates in Anhui, China	18–20 February 2020	Convenience sampling	840	276 (32.9)	Mean: 20.2	Online self-administered questionnaire	SDS ⩾ 53	233 (27.7)
Wu *et al*. (2021)	Undergraduates in 16 provinces and cities in China	4–12 February 2020	Random sampling	11 787	5056 (42.9)	Mean: 20.5	Online self-administered questionnaire	PHQ-9 ⩾ 5	3053 (25.9)
Yu *et al*. (2021)	Undergraduates in China	3–15 March 2020	Convenience sampling	1681	592 (35.2)	NR	Online self-administered questionnaire	CES-D ⩾ 16	955 (56.8)

NR, not reported; s.d., standard deviation; PHQ-9, 9-item Patient Health Questionnaire; DASS-21, Depression, Anxiety and Stress Scale – 21 Items; PQEEPH, Psychological Questionnaires for Emergent Events of Public Health; SCL-90-R, Symptom Checklist-90-Revised; CES-D, Center for Epidemiologic Studies Depression Scale; SDS, Zung's Self-Depression Rating Scale; HADS, Hospital Anxiety and Depression Scale.

### RoB of included studies

In total, 31 studies had a RoB score of ‘0–3’, 42 had a RoB score of ‘4–6’ and 11 had a RoB score of ‘7–8’. No study was scored nine. The two most common methodological issues were inappropriate sample frame (*n* = 62) and problematic sampling method (*n* = 58) (online Supplementary Table 2).

### Meta-analysis of prevalence of depressive symptoms

**Fig. 2.**
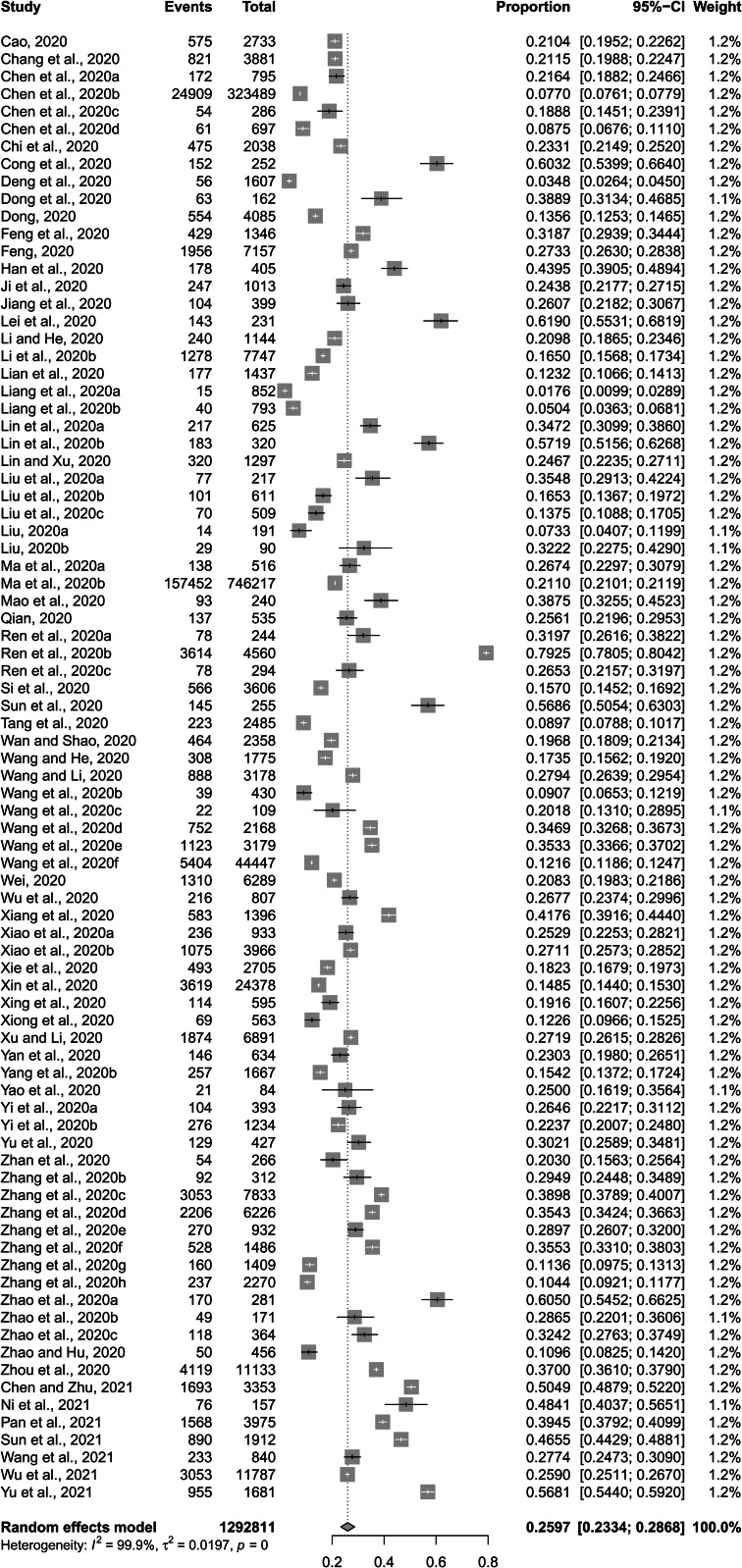
Forest plot of prevalence of depressive symptoms among Chinese university students amid the COVID-19 pandemic.

**Fig. 3.**
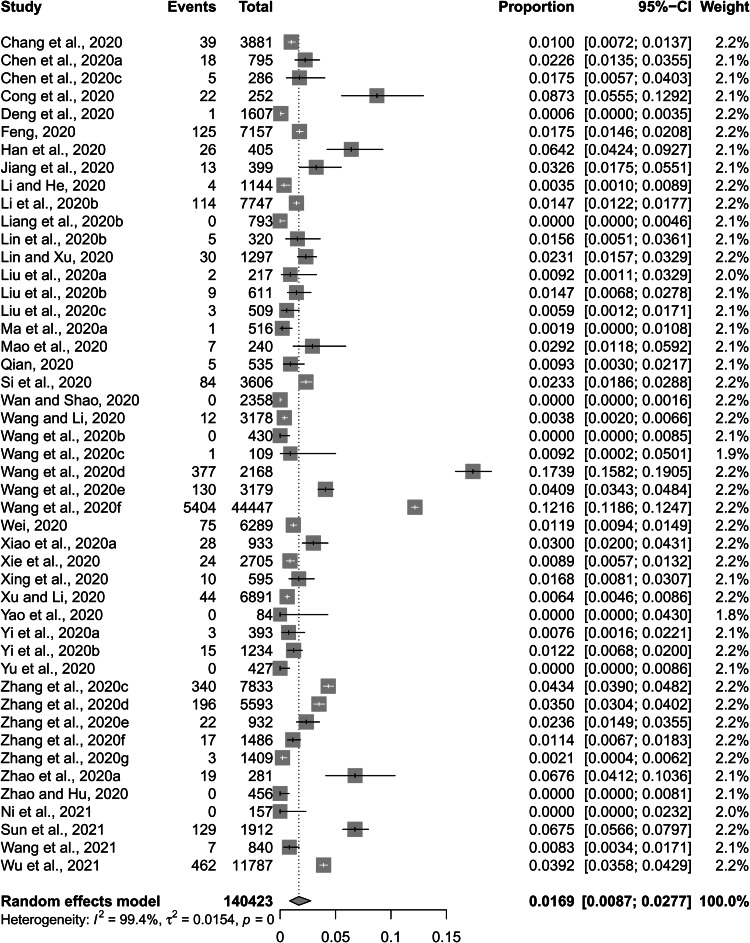
Forest plot of prevalence of severe depressive symptoms among Chinese university students amid the COVID-19 pandemic.

The pooled prevalence of depressive symptoms among Chinese university students was 26.0% (%CI: 23.3–28.9%) (Fig. 2). Pooled prevalence rate of severe depressive symptoms was 1.69% (95%CI: 0.87–2.77%) (Fig. 3).

The combined prevalence rates of depressive symptoms were significantly higher in female than in male students (30.8% *v*. 28.6%, *p* < 0.001), in students with siblings than in only child students (24.2% *v*. 20.7%, *p* < 0.001), in overseas than in domestic students (44.5% *v*. 25.6%, *p* < 0.001), in postgraduates than in undergraduates (29.3% *v*. 22.9%, *p* < 0.001), in students living in Hubei than in those living in provinces other than Hubei (27.5% *v*. 22.3%, *p* < 0.001), in students from universities of Hubei than in those from universities of other provinces (26.2% *v*. 23.1%, *p* < 0.001), in students who were in close contact with COVID-19 than in those who had no history of COVID-19 contact (46.0% *v*. 25.0%, *p* < 0.001), and in students who had friends, classmates or relatives infected with COVID-19 than in those who did not (39.7% *v*. 24.0%, *p* < 0.001) (Table 2).

**Table 2.** Results of meta-analyses of prevalence of depressive symptoms among Chinese university students

Subpopulation by variable	Number of studies	Sample size	Number of depressed students	Heterogeneity, I2 (%) (P)	Pooled prevalence (95%CI), %	Proportion transformation approach	*Z*	*P*	Cohen's d^a^
Overall									
Depressive symptoms	84	1 292 811	235 330	99.9 (<0.001)	26.0 (23.3, 28.9)	Freeman−Tukey double arcsine			
Severe depressive symptoms	47	140 423	7831	99.4 (<0.001)	1.69 (0.87, 2.77)	Freeman−Tukey double arcsine			
Gender									
Male	27	352 972	70 616	99.8 (<0.001)	28.6 (21.4, 38.3)	Log			
Female	28	449 308	106 677	99.9 (<0.001)	30.8 (24.3, 39.2)	Log	119.34	<0.001	0.270
Ethnic group									
Han	2	3079	565	99.4 (<0.001)	21.0 (0.64, 41.3)	Untransformed			
Minorities	2	537	101	97.9 (<0.001)	21.7 (0.00, 46.4)	Untransformed	0.66	0.321	0.032
Residence place									
Urban	11	37 382	9796	99.9 (<0.001)	27.4 (18.0, 41.8)	Log			
Rural	11	47 872	10 487	99.9 (<0.001)	26.8 (16.3, 44.2)	Log	6.41	<0.001	0.044
The only-child in the family									
Yes	4	1931	309	98.3 (<0.001)	20.7 (9.81, 43.6)	Log			
No	4	2194	419	99.2 (<0.001)	24.2 (10.4, 56.5)	Log	5.53	<0.001	0.171
Subject category									
Medical	34	33 263	10 717	99.5 (<0.001)	27.5 (21.0, 34.6)	Arcsine			
Non-medical	20	32 329	9181	99.7 (<0.001)	27.5 (20.6, 36.7)	Log	<0.001	0.399	<0.001
Type of students									
Oversea students	2	423	201	97.9 (<0.001)	44.5 (13.5, 75.6)	Untransformed			
Domestic students	82	1 292 388	235 129	99.9 (<0.001)	25.6 (22.9, 28.3)	Freeman−Tukey double arcsine	12.32	<0.001	0.844
Grade									
Undergraduates	42	1 183 315	206 398	99.9 (<0.001)	22.9 (19.6, 26.3)	Freeman−Tukey double arcsine			
Postgraduates	14	20 303	4192	99.1 (<0.001)	29.3 (21.6, 37.7)	Arcsine	112.14	<0.001	1.028
Geographic location of current residence									
Hubei	9	14 849	3977	95.0 (<0.001)	27.5 (22.8, 32.2)	Untransformed			
Non-Hubei	22	768 375	162 354	99.5 (<0.001)	22.3 (17.6, 27.9)	Logit	130.35	<0.001	1.033
Location of the university									
Hubei	8	23 290	6455	99.3 (<0.001)	26.2 (19.5, 33.6)	Freeman−Tukey double arcsine			
Non-Hubei	59	1 190 886	207 920	99.9 (<0.001)	23.1 (19.4, 27.2)	Logit	66.74	<0.001	0.543
Close contact with COVID-19-infected persons									
Yes	3	831	382	36.0 (0.2097)	46.0 (42.6, 49.4)	Arcsine			
No	3	13 504	4244	99.8 (<0.001)	25.0 (6.00, 44.1)	Untransformed	101.75	<0.001	1.500
Having friends, classmates or relatives infected with COVID-19									
Yes	6	11 002	3973	89.0 (<0.001)	39.7 (32.6, 48.4)	Log			
No	6	787 131	161 347	99.8 (<0.001)	24.0 (17.5, 32.9)	Log	202.82	<0.001	1.973

a Because sample sizes of different cohorts are very large, a statistically significant difference between two cohorts does not guarantee a clinical significant difference. To indicate the actual difference between two cohorts, Cohen's *d* was additionally calculated to assess the magnitude of the difference between the two rates, with 0.20–0.49, 0.50–0.79 and 0.80 and above being considered as small, medium and large actual differences, respectively. In the main text, we only reported the comparison results of different cohorts with Cohen's *d* values of approximately 0.20 or higher.

### Publication bias among included studies

**Fig. 4.**
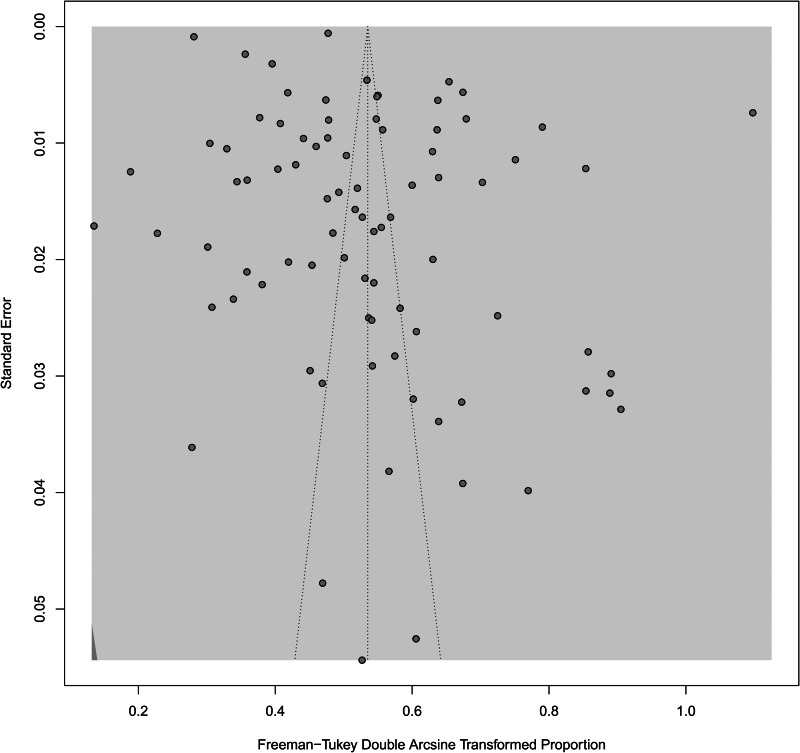
Funnel plot of publication bias among the 84 included studies.

As shown in Fig. 4, the funnel plot was generally symmetric. The *p* value of the Begg's test was 0.169. No statistically significant publication bias was detected across the 84 included studies.

### Source of heterogeneity

Five factors were identified as sources of heterogeneity across included studies (Table 3): survey period, % of male students among the total sample, scale of depressive symptoms, cutoff score of the scale of depressive symptoms and level of RoB. Specifically, significantly higher pooled prevalence rates of depressive symptoms were observed in studies conducted during the late stage of the COVID-19 outbreak than in those conducted during the early stage (31.0% *v*. 21.8%, *p* = 0.015), in studies with a percentage of males <50% than in those with a percentage of males ⩾50% (27.3% *v*. 20.6%, *p* = 0.033), in studies assessing depressive symptoms with CES-D than in those using SCL-90-R (40.0% *v*. 11.5%, *p* = 0.002), in studies defining the presence of depressive symptoms as ‘PHQ-9 ⩾ 5’ than in those defining it as ‘PHQ-9 ⩾ 10’ (29.2% *v*. 15.5%, *p* < 0.001), and in studies with a high RoB than in those with a low RoB (28.4% *v*. 20.6%, *p* = 0.011).

**Table 3.** Subgroup analysis of the source of heterogeneity of included studies

Study characteristics	Number of studies	Sample size	Number of depressed students	Heterogeneity, I2 (%) (P)	Pooled prevalence (95% CI), %	*Q*	*P*
Survey period							
Early stage of COVID-19 outbreak	32	1 214 858	211 065	99.9 (<0.001)	21.8 (18.3, 25.5)	Reference	
Late stage of COVID-19 outbreak	34	50 290	18 286	99.6 (<0.001)	31.0 (24.5, 38.0)	5.93	0.015
Post-COVID-19 era	6	12 881	3411	99.6 (<0.001)	28.9 (15.7, 44.2)	0.95	0.329
Not reported	12	14 782	2568	98.2 (<0.001)	22.3 (17.2, 27.8)	0.03	0.869
Percentage of males among the survey sample							
⩾50%	14	22 866	4321	98.8 (<0.001)	20.6 (15.7, 26.0)	Reference	
<50%	65	1 262 658	229 437	99.9 (<0.001)	27.3 (24.2, 30.5)	4.53	0.033
Not reported	5	7287	1572	96.3 (<0.001)	24.1 (15.5, 34.0)	0.45	0.500
Mean/median age (years)							
>20.8	19	70 547	16 696	99.9 (<0.001)	31.4 (20.2, 43.8)	Reference	
⩽20.8	21	83 904	19 037	99.5 (<0.001)	25.7 (21.4, 30.2)	0.81	0.367
Not reported	44	1 138 360	199 597	99.9 (<0.001)	23.9 (20.5, 27.5)	1.49	0.222
Survey method							
Online self-administered	77	1 289 363	234 634	99.9 (<0.001)	26.3 (23.5, 29.1)		
Paper−pencil self-administered	7	3448	696	95.6 (<0.001)	22.3 (15.7, 29.5)	1.05	0.306
Sampling method							
Convenience sampling	61	1 170 724	213 585	99.9 (<0.001)	27.1 (23.7, 30.7)		
Probability sampling	23	122 087	21 745	99.4 (<0.001)	23.0 (19.7, 26.4)	2.87	0.090
Assessment							
Depression subscale of SCL-90-R	8	9378	1215	98.4 (<0.001)	11. 5 (6.6, 17.5)	Reference	
PHQ-9	37	1 189 597	212 581	99.9 (<0.001)	27.3 (23.5, 31.2)	17.39	<0.001
CES-D	7	56 504	10 734	99.9 (<0.001)	40.0 (22.4, 58.9)	10.07	0.002
Depression subscale of PQEEPH	2	3442	565	57.3 (0.130)	16.4 (14.5, 18.3)	2.42	0.120
Depression subscale of DASS-21	7	7055	1119	98.9 (<0.001)	21.1 (11.7, 32.5)	2.81	0.094
SDS	22	26 201	8970	99.7 (<0.001)	28.4 (19.0, 38.9)	9.25	0.002
Depression subscale of HADS	1	634	146	Not applicable	23.0 (19.8, 26.4)	10.45	0.001
Cut-off score of PHQ-9							
⩾10	5	352 561	29 275	99.4 (<0.001)	15.5 (10.3, 21.5)		
⩾5	32	837 036	183 306	99.8 (<0.001)	29.2 (26.4, 32.1)	15.33	<0.001
RoB score							
7–8 (low)	11	95 842	15 507	99.5 (<0.001)	20.6 (16.4, 25.0)	Reference	
4–6 (moderate)	42	1 156 633	208 692	99.9 (<0.001)	25.7 (21.7, 29.9)	2.88	0.090
0–3 (high)	31	40 336	11 131	98.8 (<0.001)	28.4 (24.3, 32.7)	6.49	0.011

PHQ-9, 9-item Patient Health Questionnaire; DASS-21, Depression, Anxiety and Stress Scale – 21 Items; PQEEPH, Psychological Questionnaires for Emergent Events of Public Health; SCL-90-R, Symptom Checklist-90-Revised; CES-D, Center for Epidemiologic Studies Depression Scale; SDS, Zung's Self-Depression Rating Scale; HADS, Hospital Anxiety and Depression Scale.

## Discussion

### Main findings

This systematic review and meta-analysis summarised studies estimating the prevalence of depressive symptoms among Chinese university students amid the COVID-19 pandemic. We found an overall prevalence rate of 26.0% of depressive symptoms in Chinese university students and significantly higher rates in female students (*v*. males), in students with siblings (*v*. only children), in overseas students (*v*. domestic), in postgraduates (*v*. undergraduates), in students living within the COVID-19 epicentre (*v*. those living outside), in students from universities at the epicentre (*v*. those from universities of provinces other than Hubei), in close contacts of COVID-19-infected persons (*v*. those without a history of COVID-19 contact) and in students who had COVID-19-infected friends, classmates or relatives (*v*. those who did not). In addition, 1.69% of Chinese university students had severe depressive symptoms.

Compared to the 23.8% prevalence of depressive symptoms among Chinese university students during the non-COVID-19 era (Lei *et al*., 2016), a higher prevalence of depressive symptoms (26.0%) was found in Chinese university students amid the COVID-19 pandemic. Nevertheless, the absolute difference between the two rates (2.2%) is not very large in magnitude. We argue that the result from this direct comparison should be considered with caution because of the significant heterogeneity in the methodologies of included studies. As shown in Table 3, the pooled prevalence of depressive symptoms rose to 29.2% when included studies were restricted to those defining the presence of depressive symptoms as ‘PHQ-9 ⩾ 5’. Previously, empirical studies have reported that the prevalence rates of depressive symptoms in Chinese university students were 19.2% (PHQ-9 ⩾ 5), 7.8–12.6% (PHQ-9 ⩾ 10) and 26.9% (CES-D ⩾ 16) (He *et al*., 2014; Wu, 2019; Zhao *et al*., 2019; Gao *et al*., 2020*b*; Leung *et al*., 2020; Li *et al*., 2021), which are all lower than the corresponding figures in our study (29.2%, 15.5% and 40.0%, Table 3). Moreover, the 1.69% prevalence of severe depressive symptoms in our study was higher than that reported in two previous studies with samples of Chinese university students (0.5–0.9%) (Ma *et al*., 2019; Zhao *et al*., 2019). These data suggest an elevated risk of depressive symptoms in Chinese university students during the COVID-19 pandemic.

In addition to the abovementioned postponement of graduation, home quarantine and social disconnectedness due to the COVID-19 pandemic, the cooccurring ‘infodemic’ may also explain the elevated risk of depressive symptoms in university students. This is because smartphone and social media use are very popular among Chinese university students, and students are more likely to be exposed to negative information or even rumours from social media platforms such as short videos of overcrowded hospitals, physically and emotionally exhausted physicians and helpless infected patients. As a supporting case, in this pandemic, Chinese researchers have found the significant association between frequent social media exposure and depressive symptoms in the general population (Gao *et al*., 2020*a*).

### Cohort-specific prevalence of depressive symptoms

The higher risk of depressive symptoms in female than in male students during the COVID-19 pandemic is in line with the findings of previous studies with samples of general university students (Li *et al*., 2018; Gao *et al*., 2020*b*; Ismail *et al*., 2020). This phenomenon could be ascribed to the personality traits of females, such as higher levels of neuroticism/negative emotionality and conscientiousness, in comparison to males (Klein *et al*., 2011; Weisberg *et al*., 2011). A meta-analysis of studies comparing the psychopathology between only children and children with siblings in China revealed the small mental health advantage experienced by only child university students in comparison to their peers with siblings, i.e. fewer psychiatric symptoms, including depressive symptoms (Falbo and Hooper, 2015). It seems that this phenomenon also exists in university students affected by the COVID-19 pandemic, i.e. significantly lower rate of depressive symptoms in only child students than in students with siblings, with a small magnitude of difference between the two groups (Cohen's *d* = 0.17) (Table 2).

One possible explanation for the higher risk of depressive symptoms in overseas than in domestic students is the status of ethnic minority groups in foreign countries (Li *et al*., 2014). As migrants, overseas students per se have inadequate social support, and this situation worsens owing to the social distancing requirements during the COVID-19 pandemic, potentially increasing the risk of depressive symptoms (Zhong *et al*., 2015). Due to the higher levels of academic stress in postgraduates than in undergraduates, it is generally believed that postgraduates are at higher risk for depressive symptoms than undergraduates in China (Wang *et al*., 2019). Similarly, a significantly higher prevalence of depressive symptoms in postgraduates than in undergraduates was observed in our study. According to our experiences with some university students from the crisis hotline services during the outbreak period, the negative impact of the COVID-19 pandemic on academic achievement is greater in postgraduates than in undergraduates since undergraduates are able to continue their studies through online courses, but many postgraduates rely on university campus labs to continue their research. Because of the closure of campuses, postgraduates are more likely to be depressed.

Due to Hubei residents’ higher risk of infection and province-wide stringent mass quarantine measures, an elevated risk of depressive symptoms in students living in the epicentre relative to that in students living outside the epicentre is expected. Despite having left Hubei before the Spring Festival, students from universities in Hubei had been compulsorily isolated for medical observation in their hometowns and experienced a high level of discrimination and social exclusion due to their potential to spread the COVID-19 virus at the initial stage of the outbreak (He *et al*., 2020). Therefore, it is reasonable to find significantly higher rates of depressive symptoms in students from universities at the epicentre than in those from universities of provinces other than Hubei in our study.

Studies have reported the significant association of depressive symptoms with having relatives or acquaintances infected with COVID-19 in general populations of both China and Italy during the COVID-19 pandemic (Mazza *et al*., 2020; Zhong *et al*., 2020). Consistent with these findings, the rate of depressive symptoms was significantly higher in university students with COVID-19-infected acquaintances or relatives, which may be attributed to these students’ high levels of concern about the health of the infected persons. Previous studies have found a greater level of fear of COVID-19 infection in persons who were suspected of having COVID-19, which was in turn associated with a higher risk of depressive symptoms (Koçak *et al*., 2021; Tsang *et al*., 2021). For a similar reason, university students with a history of COVID-19 contact exhibited a significantly higher prevalence of depressive symptoms.

### Findings from subgroup analysis

Subgroup analysis revealed a higher prevalence of depressive symptoms in studies with samples with fewer men, which is consistent with the female predominance phenomenon of depression (Albert, 2015). However, what is counterintuitive is the higher risk of depressive symptoms in studies conducted late in the COVID-19 outbreak than that in studies conducted early in the COVID-19 outbreak in the subgroup analysis because the daily number of newly confirmed COVID-19 cases in China peaked during the early stage, and the outbreak was under control during the late stage. Similarly, a two-wave longitudinal study in China found increased severity of depressive symptoms in a cohort of the general population four weeks after the epidemic's peak relative to the initial COVID-19 outbreak (Wang *et al*., 2020*a*). We speculate that during the early stage, people may have been shocked by the sudden outbreak, and they focused on safety and physical health. After the outbreak, the negative impacts of the pandemic, including economic loss and unemployment, gradually increased with time, leading people to feel depressed. Because of the problematic methodology of poorly designed studies, i.e. mental health surveys adopting convenience sampling are likely to recruit students having potential needs for mental health services, a statistically higher prevalence of depressive symptoms in studies with a high level of RoB was found in this study.

### Limitations

This study has some limitations. First, none of the included studies were rated as completely low RoB. Subgroup analysis according to RoB level found a significantly higher prevalence of depressive symptoms in studies with a high level of RoB, so it is possible that the reported overall pooled estimate overestimates the true prevalence. Second, because several included studies used strict criteria to define the presence of depressive symptoms (i.e. PHQ-9 ⩾ 10), we may have underestimated the prevalence of depressive symptoms. Given the above two limitations, it is difficult to assess the magnitude and direction of bias in the prevalence estimate. Cautions are needed when generalising our findings. Third, even after stratifying the studies, high levels of heterogeneity were still kept within each strata of study in the subgroup analysis, so there remained other factors associated with the risk of depressive symptoms that were not identified. The heterogeneity of the results suggests that further rigorously designed studies using widely accepted assessments of depressive symptoms and representative samples of Chinese university students amid the COVID-19 pandemic are warranted to arrive at accurate estimates. Fourth, because of the small number of studies during the postoutbreak period, longitudinal data are needed to examine the trajectory of depressive symptoms in Chinese university students in the postpandemic era. Fifth, since the sample size of overseas students was relatively small (*n* = 423), the sample representativeness of overseas students may be limited in our study. Finally, patterns of utilisation of mental health services among depressed students are very important for mental health planning and policy-making in the context of the COVID-19 pandemic, but the included studies provided little information on service use.

### Implications and conclusions

In this study, over one out of every four Chinese university students had depressive symptoms, which suggests a high level of mental healthcare need in this population amid the COVID-19 pandemic. Depression takes a high toll on individuals, families and societies, and, in particular, it is a major risk factor for attempted and completed suicide. Given the high prevalence of depressive symptoms, mental health services for this population amid the pandemic should include periodic evaluation of depressive symptoms to ensure early identification of students with severe depressive symptoms or high risk of suicide and psychiatric assessment and treatment when necessary. The higher prevalence rates of depressive symptoms revealed in several cohorts of Chinese university students (i.e. postgraduates, students living in the epicentre and COVID-19 contacts) indicate that cohort-specific prevention programmes, which are probably cost-effective, need to be designed.

China is a mental health services resource-poor country, so university managers and staff, including campus psychological counselors, should have a critical role in depression prevention; for example, they could provide expanded social support to students at risk, engage in follow-up care, mental health education and periodic screening of depressed students and promote social connectedness between students. Although the pandemic increases physical distances between staff and students, support services can be easily provided to students via smartphones.

In addition, the 28.9% prevalence of depressive symptoms during the postoutbreak era in this study (Table 3) and some small new COVID-19 outbreaks in recent months in China suggest the necessity of continuous mental health monitoring and services for Chinese university students during the postoutbreak era. Further rigorous research is also needed to understand the longitudinal changes in depressive symptoms of Chinese university students during the postoutbreak era.

## Data Availability

All the data involved have been included in Tables and Figures of this paper, including supplementary files.

## Supplementary material

For supplementary material accompanying this paper visit http://dx.doi.org/10.1017/S2045796021000202. click here to view supplementary material
