# Supplementary material for: Prevalence of depressive symptoms among Chinese university students amid the COVID-19 pandemic: a systematic review and meta-analysis
Source: Epidemiol Psychiatr Sci. 2021 Mar 26;30:e31. doi: 10.1017/S2045796021000202 (PMC8047400; doi:10.1017/S2045796021000202)
Supplement: Supplementary file 1 [file epssup.zip › S2045796021000202sup004.docx]

**Supplementary Table 2. Risk of bias appraisal of included studies**

| Study | Q1 | Q2 | Q3 | Q4 | Q5 | Q6 | Q7 | Q8 | Q9 | Risk of bias score |
| --- | --- | --- | --- | --- | --- | --- | --- | --- | --- | --- |
| ([Cao, 2020](#_ENREF_4)) | No | No | Yes | No | Unclear | Yes | Unclear | Yes | Unclear | 3 |
| ([Chang et al., 2020](#_ENREF_5)) | No | No | Yes | Yes | Unclear | Yes | Unclear | Yes | Unclear | 4 |
| ([Chen et al., 2020a](#_ENREF_7)) | Unclear | Unclear | Yes | Yes | Unclear | Yes | Unclear | No | Unclear | 3 |
| ([Chen et al., 2020b](#_ENREF_8)) | Yes | No | Yes | Yes | No | Yes | Yes | Yes | Unclear | 6 |
| ([Chen et al., 2020c](#_ENREF_9)) | No | No | No | No | Unclear | Yes | Unclear | Yes | Unclear | 2 |
| ([Chen et al., 2020d](#_ENREF_10)) | Yes | Yes | Yes | No | Yes | Yes | Unclear | Yes | Yes | 7 |
| ([Chi et al., 2020](#_ENREF_11)) | No | No | Yes | Yes | Unclear | Yes | Unclear | Yes | Unclear | 4 |
| ([Cong et al., 2020](#_ENREF_12)) | No | No | No | No | Unclear | Yes | Unclear | Yes | Unclear | 2 |
| ([Deng et al., 2020](#_ENREF_14)) | No | No | Yes | Yes | Unclear | Yes | Unclear | Yes | Unclear | 4 |
| ([Dong et al., 2020](#_ENREF_16)) | No | Yes | No | Yes | Yes | Yes | Unclear | Yes | Yes | 6 |
| ([Dong, 2020](#_ENREF_17)) | Yes | Yes | Yes | No | Yes | Yes | Unclear | Yes | Yes | 7 |
| ([Feng et al., 2020](#_ENREF_21)) | Unclear | Unclear | Yes | Yes | Yes | Yes | Unclear | Yes | Yes | 6 |
| ([Feng, 2020](#_ENREF_20)) | Yes | Yes | Yes | Yes | Unclear | Yes | Unclear | Yes | Yes | 7 |
| ([Han et al., 2020](#_ENREF_24)) | No | No | Yes | No | Unclear | Yes | Yes | Yes | Unclear | 4 |
| ([Ji et al., 2020](#_ENREF_29)) | Yes | Yes | Yes | Yes | Unclear | Yes | Unclear | Yes | Unclear | 6 |
| ([Jiang et al., 2020](#_ENREF_30)) | Yes | Yes | Yes | No | Yes | Yes | Yes | Yes | Yes | 8 |
| ([Lei et al., 2020](#_ENREF_34)) | No | No | No | No | Unclear | Yes | Unclear | Yes | Yes | 3 |
| ([Li and He, 2020](#_ENREF_39)) | No | No | Yes | No | Unclear | Yes | Unclear | Yes | Unclear | 3 |
| ([Li et al., 2020b](#_ENREF_42)) | Yes | No | Yes | Yes | No | Yes | Unclear | Yes | No | 5 |
| ([Lian et al., 2020](#_ENREF_43)) | No | Yes | Yes | No | Yes | Yes | Unclear | Yes | Yes | 6 |
| ([Liang et al., 2020a](#_ENREF_44)) | No | No | Yes | No | Yes | No | No | Yes | Yes | 4 |
| ([Liang et al., 2020b](#_ENREF_45)) | No | No | Yes | No | Yes | Yes | Unclear | No | Yes | 4 |
| ([Lin et al., 2020a](#_ENREF_46)) | No | No | Yes | No | Unclear | Yes | Unclear | Yes | Unclear | 3 |
| ([Lin et al., 2020b](#_ENREF_48)) | No | No | Yes | No | Unclear | Yes | Yes | Yes | Unclear | 4 |
| ([Lin and Xu, 2020](#_ENREF_47)) | No | No | Yes | No | Unclear | Yes | Unclear | Yes | Unclear | 3 |
| ([Liu et al., 2020a](#_ENREF_50)) | No | No | No | Yes | No | Yes | Unclear | Yes | No | 3 |
| ([Liu et al., 2020b](#_ENREF_53)) | No | No | Yes | No | Yes | Yes | Unclear | Yes | Yes | 5 |
| ([Liu et al., 2020c](#_ENREF_54)) | No | No | Yes | Yes | Unclear | Yes | Unclear | Yes | Unclear | 4 |
| ([Liu, 2020a](#_ENREF_51)) | Unclear | Unclear | No | No | Yes | Yes | Unclear | Yes | Yes | 4 |
| ([Liu, 2020b](#_ENREF_55)) | No | Yes | No | No | Yes | Yes | Unclear | Yes | Yes | 5 |
| ([Ma et al., 2020a](#_ENREF_58)) | Unclear | Unclear | Yes | Yes | Yes | Yes | Unclear | Yes | Yes | 6 |
| ([Ma et al., 2020b](#_ENREF_59)) | Yes | No | Yes | Yes | Unclear | Yes | Unclear | Yes | Unclear | 5 |
| ([Mao et al., 2020](#_ENREF_60)) | No | No | No | Yes | Yes | Yes | Unclear | Yes | Yes | 5 |
| ([Qian, 2020](#_ENREF_66)) | No | No | Yes | Yes | Unclear | Yes | Unclear | Yes | Unclear | 4 |
| ([Ren et al., 2020a](#_ENREF_67)) | No | No | No | No | Unclear | Unclear | Unclear | Unclear | Unclear | 0 |
| ([Ren et al., 2020b](#_ENREF_69)) | No | No | Yes | Yes | Unclear | Yes | Unclear | Yes | Unclear | 4 |
| ([Ren et al., 2020c](#_ENREF_70)) | No | No | No | Yes | Unclear | Yes | Yes | Yes | Unclear | 4 |
| ([Si et al., 2020](#_ENREF_72)) | No | No | Yes | Yes | Unclear | Yes | Unclear | Yes | Unclear | 4 |
| ([Sun et al., 2020](#_ENREF_74)) | No | No | No | Yes | Unclear | Yes | Unclear | Yes | Yes | 4 |
| ([Tang et al., 2020](#_ENREF_75)) | No | No | Yes | Yes | No | Yes | Unclear | Yes | No | 4 |
| ([Wan and Shao, 2020](#_ENREF_79)) | No | No | Yes | No | Unclear | Yes | Unclear | Yes | Unclear | 3 |
| ([Wang and He, 2020](#_ENREF_82)) | No | No | Yes | No | Unclear | Yes | Unclear | Yes | Unclear | 3 |
| ([Wang and Li, 2020](#_ENREF_88)) | No | No | Yes | No | Unclear | Yes | Unclear | Yes | Unclear | 3 |
| ([Wang et al., 2020b](#_ENREF_83)) | Unclear | Unclear | Yes | Yes | Unclear | Yes | Unclear | No | Unclear | 3 |
| ([Wang et al., 2020c](#_ENREF_84)) | No | No | No | No | Unclear | Yes | Unclear | Yes | Unclear | 2 |
| ([Wang et al., 2020d](#_ENREF_86)) | No | No | Yes | Yes | Unclear | Yes | Unclear | Yes | Unclear | 4 |
| ([Wang et al., 2020e](#_ENREF_87)) | No | No | Yes | No | Unclear | Yes | Unclear | Yes | Unclear | 3 |
| ([Wang et al., 2020f](#_ENREF_89)) | Yes | Yes | Yes | Yes | Yes | No | Unclear | Yes | Yes | 7 |
| ([Wei, 2020](#_ENREF_90)) | Unclear | Unclear | Yes | No | Unclear | Unclear | Unclear | Unclear | Unclear | 1 |
| ([Wu et al., 2020](#_ENREF_92)) | No | Yes | Yes | No | Unclear | Yes | Unclear | Yes | Unclear | 4 |
| ([Xiang et al., 2020](#_ENREF_95)) | No | No | Yes | Yes | No | Yes | Unclear | Yes | No | 4 |
| ([Xiao et al., 2020a](#_ENREF_96)) | Yes | Yes | Yes | Yes | Yes | Yes | Unclear | Yes | Yes | 8 |
| ([Xiao et al., 2020b](#_ENREF_97)) | Yes | Yes | Yes | No | Unclear | Yes | Unclear | Yes | Unclear | 5 |
| ([Xie et al., 2020](#_ENREF_98)) | No | No | Yes | No | Unclear | Yes | Unclear | Yes | Unclear | 3 |
| ([Xin et al., 2020](#_ENREF_99)) | Yes | Yes | Yes | Yes | Yes | Yes | Unclear | Yes | Yes | 8 |
| ([Xing et al., 2020](#_ENREF_100)) | Unclear | No | Yes | No | Yes | Yes | Unclear | Yes | Yes | 5 |
| ([Xiong et al., 2020](#_ENREF_101)) | No | No | Yes | Yes | Unclear | Yes | Unclear | Yes | Unclear | 4 |
| ([Xu and Li, 2020](#_ENREF_102)) | No | Yes | Yes | No | Unclear | Yes | Unclear | Yes | Unclear | 4 |
| ([Yan et al., 2020](#_ENREF_103)) | No | Yes | Yes | Yes | No | Yes | Yes | Yes | Yes | 7 |
| ([Yang et al., 2020b](#_ENREF_105)) | No | No | Yes | Yes | Unclear | Yes | Yes | Yes | Unclear | 5 |
| ([Yao et al., 2020](#_ENREF_106)) | No | No | No | Yes | Unclear | Yes | Unclear | Yes | Unclear | 3 |
| ([Yi et al., 2020a](#_ENREF_107)) | No | Yes | Yes | Yes | Yes | Yes | Unclear | Yes | Yes | 7 |
| ([Yi et al., 2020b](#_ENREF_108)) | No | No | Yes | No | Unclear | Yes | Unclear | Yes | Unclear | 3 |
| ([Yu et al., 2020](#_ENREF_109)) | No | No | Yes | No | Unclear | Yes | Unclear | Yes | Unclear | 3 |
| ([Zhan et al., 2020](#_ENREF_111)) | No | No | No | Yes | Unclear | Yes | Unclear | Yes | Unclear | 3 |
| ([Zhang et al., 2020b](#_ENREF_113)) | No | No | No | Yes | Unclear | Yes | Unclear | Yes | Unclear | 3 |
| ([Zhang et al., 2020c](#_ENREF_114)) | No | No | Yes | Yes | Unclear | Yes | Unclear | Yes | Unclear | 4 |
| ([Zhang et al., 2020d](#_ENREF_115); [Yang et al., 2020a](#_ENREF_104)) | No | No | Yes | Yes | Unclear | Yes | Unclear | Yes | Unclear | 4 |
| ([Zhang et al., 2020e](#_ENREF_116)) | No | Yes | Yes | Yes | Yes | Yes | Unclear | Yes | Yes | 7 |
| ([Zhang et al., 2020f](#_ENREF_117)) | Unclear | Unclear | Yes | Yes | Unclear | Yes | Unclear | Yes | Unclear | 4 |
| ([Zhang et al., 2020g](#_ENREF_118)) | No | No | Yes | No | Unclear | Yes | Unclear | Yes | Unclear | 3 |
| ([Zhang et al., 2020h](#_ENREF_119)) | No | No | Yes | Yes | Unclear | Yes | Unclear | Yes | Unclear | 4 |
| ([Zhao et al., 2020a](#_ENREF_120)) | No | No | No | Yes | Unclear | Yes | Unclear | Yes | Unclear | 3 |
| ([Zhao et al., 2020b](#_ENREF_121)) | No | No | No | No | Unclear | Yes | Unclear | Yes | Unclear | 2 |
| ([Zhao et al., 2020c](#_ENREF_123)) | No | No | Yes | No | Unclear | Yes | Unclear | Yes | Unclear | 3 |
| ([Zhao and Hu, 2020](#_ENREF_125)) | Unclear | Unclear | Yes | No | Unclear | Yes | Unclear | Yes | Unclear | 3 |
| ([Zhou et al., 2020](#_ENREF_128)) | No | No | Yes | No | Unclear | Yes | Unclear | Yes | Yes | 4 |
| ([Chen and Zhu, 2021](#_ENREF_6)) | No | No | Yes | Yes | Unclear | Yes | Unclear | No | Unclear | 3 |
| ([Ni et al., 2021](#_ENREF_64)) | No | No | No | No | Unclear | Yes | Unclear | Unclear | Unclear | 1 |
| ([Pan et al., 2021](#_ENREF_65)) | No | No | Yes | No | Unclear | Yes | Unclear | Yes | Unclear | 3 |
| ([Sun et al., 2021](#_ENREF_73)) | No | No | Yes | Yes | Unclear | Yes | Unclear | Yes | Unclear | 4 |
| ([Wang et al., 2021](#_ENREF_81)) | No | No | Yes | Yes | Unclear | Yes | Unclear | Yes | Unclear | 4 |
| ([Wu et al., 2021](#_ENREF_94)) | Yes | Yes | Yes | Yes | Yes | Yes | Unclear | Yes | Yes | 8 |
| ([Yu et al., 2021](#_ENREF_110)) | No | No | Yes | No | Yes | Yes | Unclear | Yes | Yes | 5 |

Note: Q1-9: 9 questions of The Joanna Briggs Institute Critical Appraisal Checklist for Studies Reporting Prevalence Data, as listed below:

Q1: Was the sample frame appropriate to address the target population?

Q2: Were study participants sampled in an appropriate way?

Q3: Was the sample size adequate?

Q4: Were the study subjects and the setting described in detail?

Q5: Was the data analysis conducted with sufficient coverage of the identified sample?

Q6: Were valid methods used for the identification of the condition?

Q7: Was the condition measured in a standard, reliable way for all participants?

Q8: Was there appropriate statistical analysis?

Q9: Was the response rate adequate, and if not, was the low response rate managed appropriately?

For Q3, the minimum sample size required was 314, which was determined assuming a Type I error of 0.05, an estimated prevalence of 26.0%, and a confidence interval width equal to 0.10.
