# Supplementary material for: Prevalence of depressive symptoms among Chinese university students amid the COVID-19 pandemic: a systematic review and meta-analysis
Source: Epidemiol Psychiatr Sci. 2021 Mar 26;30:e31. doi: 10.1017/S2045796021000202 (PMC8047400; doi:10.1017/S2045796021000202)
Supplement: Supplementary file 1 [file epssup.zip › S2045796021000202sup003.doc]

**Supplementary Table 1. Literature search strategies**

| Databases | Search strategies | Publication date |
| --- | --- | --- |
| Chinese language |  |  |
| China National Knowledge Infrastructure | (AB='大学生' OR AB='学生' OR AB='青少年' OR AB='少年' OR AB='成年早期' OR AB='研究生' OR AB=‘本科生’ OR AB='专科生' OR AB='高职生' OR AB='护生' OR AB='护理实习生') AND AB='抑郁' AND (AB='肺炎' OR AB='病毒' OR AB='新冠' OR AB='冠状病毒' OR AB='疫情') | January 1, 2020 to February 10, 2021 |
| Wanfang data | 摘要:("大学生" or "学生" or"青少年" or"少年" or "成年早期" or "研究生" or "本科生" or "专科生" or "高职生" or "护生" or "护理实习生") and ("抑郁") and ("肺炎" or"病毒" or"新冠" or"冠状病毒" or "疫情") | January 1, 2020 to February 10, 2021 |
| VIP Information | (R="大学生" +"学生" +"青少年" +"少年" + "成年早期" +”研究生”+"本科生" + "专科生" + "高职生" + "护生" + "护理实习生") * R="抑郁" * (R="肺炎" +"病毒" +"新冠" +"冠状病毒" + "疫情") | January 1, 2020 to February 10, 2021 |
| English language |  |  |
| PubMed | ((("student*"[Title/Abstract]) OR ("graduate*"[Title/Abstract]) OR ("undergraduate*"[Title/Abstract]) OR ("postgraduate*"[Title/Abstract]) OR ("adolescen*"[Title/Abstract]) OR ("teen*"[Title/Abstract]) OR ("teenager*"[Title/Abstract]) OR ("youth*"[Title/Abstract]) OR ("young adult*"[Title/Abstract]) OR ("universit*"[Title/Abstract]) OR ("college*"[Title/Abstract])) AND ((Depression*[Title/Abstract]) OR (Depressive Symptom*[Title/Abstract]) OR (Emotional Depression*[Title/Abstract]) OR (Depression*, Emotional[Title/Abstract]) OR (Depressive Disorder*[Title/Abstract]) OR (Disorder*, Depressive[Title/Abstract]) OR (Neuros*, Depressive[Title/Abstract]) OR (Depressive Neuros*[Title/Abstract]) OR (Depression*, Endogenous[Title/Abstract]) OR (Endogenous Depression*[Title/Abstract]) OR (Depressive Syndrome*[Title/Abstract]) OR (Syndrome*, Depressive[Title/Abstract]) OR (Depression*, Neurotic[Title/Abstract]) OR (Neurotic Depression*[Title/Abstract]) OR (Melancholia*[Title/Abstract]) OR (Unipolar Depression*[Title/Abstract]) OR (Depression*, Unipolar[Title/Abstract]))) AND (("severe acute respiratory syndrome coronavirus 2"[Supplementary Concept]) OR ("COVID-19"[Supplementary Concept]) OR ("2019-nCoV"[Title/Abstract]) OR ("wuhan coronavirus"[Title/Abstract]) OR ("SARS-CoV-2"[Title/Abstract]) OR ("2019 novel coronavirus"[Title/Abstract]) OR ("covid 19 virus"[Title/Abstract]) OR ("coronavirus disease 2019 virus"[Title/Abstract]) OR ("covid19 virus"[Title/Abstract]) OR ("wuhan seafood market pneumonia virus"[Title/Abstract]) OR ("2019 novel coronavirus disease"[Title/Abstract]) OR ("COVID19"[Title/Abstract]) OR ("covid 19 pandemic"[Title/Abstract]) OR ("sars cov 2 infection"[Title/Abstract]) OR ("covid 19 virus disease"[Title/Abstract]) OR ("2019 novel coronavirus infection"[Title/Abstract]) OR ("2019 ncov infection"[Title/Abstract]) OR ("coronavirus disease 2019"[Title/Abstract]) OR ("coronavirus disease 19"[Title/Abstract]) OR ("2019 ncov disease"[Title/Abstract]) OR ("covid 19 virus infection"[Title/Abstract])) | January 1, 2020 to February 10, 2021 |
| Embase | ('coronavirus disease 2019'/exp OR 'severe acute respiratory syndrome coronavirus 2'/exp OR 'wuhan coronavirus':ab OR 'sars-cov-2':ab OR '2019 novel coronavirus':ab OR 'covid-19 virus':ab OR 'coronavirus disease 2019 virus':ab OR 'covid19 virus':ab OR 'wuhan seafood market pneumonia virus':ab OR '2019 novel coronavirus disease':ab OR 'covid19':ab OR 'covid-19 pandemic':ab OR 'sars-cov-2 infection':ab OR 'covid-19 virus disease':ab OR '2019 novel coronavirus infection':ab OR '2019-ncov infection':ab OR 'coronavirus disease 2019':ab OR 'coronavirus disease-19':ab OR '2019-ncov disease':ab OR 'covid-19 virus infection':ab) AND ('adolescen*':ab OR 'teenager*':ab OR 'youth*':ab OR 'student*':ab OR 'young adult*':ab OR 'undergraduate*':ab OR 'graduate*':ab OR 'postgraduate*':ab OR 'universit*':ab OR 'college*':ab OR 'teen*':ab) AND ('depression*':ab OR 'depressive disorder*':ab OR 'depressive symptom*':ab OR 'symptom*, depressive':ab OR 'emotional depression*':ab OR 'depression*, emotional':ab OR 'disorder, depressive':ab OR 'neuros*, depressive':ab OR 'depressive neuros*':ab OR 'depression*, endogenous':ab OR 'endogenous depression*':ab OR 'depressive syndrome*':ab OR 'syndrome*, depressive':ab OR 'depression*, neurotic':ab OR 'neurotic depression*':ab OR 'melancholia*':ab OR 'unipolar depression*':ab OR 'depression*, unipolar':ab) | January 1, 2020 to February 10, 2021 |
| PsycInfo | AB ( Adolescen* OR Teen* OR Teenager* OR Youth* OR Student* OR Young Adult* OR undergraduate* OR universit* OR college* OR graduate* OR postgraduate*) AND AB ( Depression* OR Depressive Symptom* OR Symptom*, Depressive OR Emotional Depression* OR Depression*, Emotional OR Depressive Disorder* OR Disorder*, Depressive OR Neuros*, Depressive OR Depressive Neuros* OR Depression*, Endogenous OR Endogenous Depression* OR Depressive Syndrome* OR Syndrome*, Depressive OR Depression*, Neurotic OR Neurotic Depression* OR Melancholia* OR Unipolar Depression* OR Depression*, Unipolar ) AND AB ( COVID-19 OR severe acute respiratory syndrome coronavirus 2 OR SARS2 OR Wuhan coronavirus OR SARS-CoV-2 OR 2019 novel coronavirus OR COVID-19 virus OR coronavirus disease 2019 virus OR COVID19 virus OR Wuhan seafood market pneumonia virus OR 2019 novel coronavirus disease OR COVID19 OR COVID-19 pandemic OR SARS-CoV-2 infection OR COVID-19 virus disease OR 2019 novel coronavirus infection OR 2019-nCoV infection OR coronavirus disease 2019 OR coronavirus disease-19 OR 2019-nCoV disease OR COVID-19 virus infection ) | January 1, 2020 to February 10, 2021 |
